# Deep sequencing and analyses of miRNAs, isomiRs and miRNA induced silencing complex (miRISC)-associated miRNome in primary human chondrocytes

**DOI:** 10.1038/s41598-017-15388-4

**Published:** 2017-11-09

**Authors:** Abdul Haseeb, Mohammad Shahidul Makki, Nazir M. Khan, Imran Ahmad, Tariq M. Haqqi

**Affiliations:** 1 Department of Anatomy and Neurobiology, North East Ohio Medical University, 4209 St. Rt. 44, Rootstown, OH 44272 USA; 2 Present Address: Department of Cellular and Molecular Medicine, Lerner Research Institute, Cleveland Clinic, Cleveland, OH USA

## Abstract

MicroRNAs, a group of small, noncoding RNAs that post-transcriptionally regulate gene expression, play important roles in chondrocyte function and in the development of osteoarthritis. We characterized the dynamic repertoire of the chondrocyte miRNome and miRISC-associated miRNome by deep sequencing analysis of primary human chondrocytes. IL-1β treatment showed a modest effect on the expression profile of miRNAs in normal and osteoarthritis (OA) chondrocytes. We found a number of miRNAs that showed a wide range of sequence modifications including nucleotide additions and deletions at 5′ and 3′ ends; and nucleotide substitutions. miR-27b-3p showed the highest expression and miR-140-3p showed the highest number of sequence variations. AGO2 RIP-Seq analysis revealed the differential recruitment of a subset of expressed miRNAs and isoforms of miRNAs (isomiRs) to the miRISC in response to IL-1β, including miR-146a-5p, miR-155-5p and miR-27b-3p. Together, these results reveal a complex repertoire of miRNAs and isomiRs in primary human chondrocytes. Here, we also show the changes in miRNA composition of the miRISC in primary human chondrocytes in response to IL-1β treatment. These findings will provide an insight to the miRNA-mediated control of gene expression in the pathogenesis of OA.

## Introduction

MicroRNAs are 18–25 nt non-coding small RNAs that post-transcriptionally regulate gene expression by inducing destabilization and degradation of specific mRNA targets and/or by repressing their translation^1^. miRNAs are first transcribed as primary transcripts (pri-miRNAs) by RNA polymerase II or polymerase III. These transcripts are further processed in the nucleus by the RNase III domain of endoribonuclease Drosha into precursor miRNA (pre-miRNA). Pre-miRNA is exported to cytoplasm and further processed by the RNase III domain of Dicer into 18–25 base pair miRNA-5p/miRNA-3p duplex. One of the strands of the duplex is embedded into Agronaute (AGO) protein to form the miRNA-induced silencing complex (miRISC)^2^. Mature miRNAs most often function as RISCs bound to the AGO proteins to modulate the expression of their target coding RNAs^3,4^. The level of AGO binding of a particular miRNA is a better indicator of its inhibitory potential compared to its total cellular expression level^5^.

Recent deep sequencing studies have revealed a complex repertoire of miRNAs with numerous types of sequence variations compared to canonical sequences annotated in miRBase. These variants are termed as isomiRs that often have different target specificities compared to their archetype counterparts^6,7^. The miRNA sequence variations include nucleotide substitutions that give rise to polymorphic isomiRs and an addition or deletion of one or more nucleotides at the 5′ and/or 3′ ends that gives rise to 5′ or 3′ isomiRs^6^. 3′ isomiRs have been reported as the most common and abundant variants^8,9^ that are thought to result from trimming, adenylation or uridylation carried out by a number of RNA modifying enzymes^8,10,11^.

Chondrocyte is the only cell-type present in cartilage and a tight control of the expression of appropriate genes in chondrocytes is crucial for cartilage function. Expression of a large number of genes is altered during OA^12^ and several miRNAs have been shown to regulate genes involved in chondrocyte function and cartilage homeostasis^13,14^. Given the importance of miRNAs in chondrocyte function and OA development it is imperative to study the complete miRNome of the chondrocytes including the expression of all the miRNA isoforms and their binding to the silencing complex.

In the present study, we performed deep sequencing analysis on chondrocytes isolated from normal as well as OA cartilage to reveal the differential expression of isomiRs. To reveal functionally active miRNAs and isomiRs, we performed an AGO2 RIP-seq analysis in primary human chondrocytes.

## Results

### General description of the small RNA-seq data

In order to determine the complete miRNome of primary human chondrocytes we employed the next generation small RNA sequencing (sRNA-Seq) approach using the Illumina platform. We prepared primary human chondrocytes from the cartilage obtained from 2 normal subjects and 3 OA patients. Primary chondrocytes were treated with IL-1β for different durations (2 hr, 12 hrs, 24 hrs). We isolated total RNA and generated a total of 20 cDNA libraries. In total, we generated 36 million reads of 20–36 bp from 20 samples with an average of 1.8 million reads per sample after filtering. On an average 85% of these sequences could be mapped to the human genome and 67% of the reads mapped to miRNAs in miRBase v21 (www.mirbase.org). Following the processing of the data, we detected 437 miRNAs annotated in the miRBase. In general, sequences obtained after pre-analysis for all libraries ranged from 20-24 nucleotides with a predominant length of 22 nucleotides.

We did not find any significant difference in the expression of the chondrocyte-expressed miRNAs across the samples. Therefore, we pooled all the samples for our analysis of sequence heterogeneities in the expressed miRNAs.

### Abundance and cumulative contribution of top-expressing miRNAs in primary human chondrocytes

**Figure 1:**
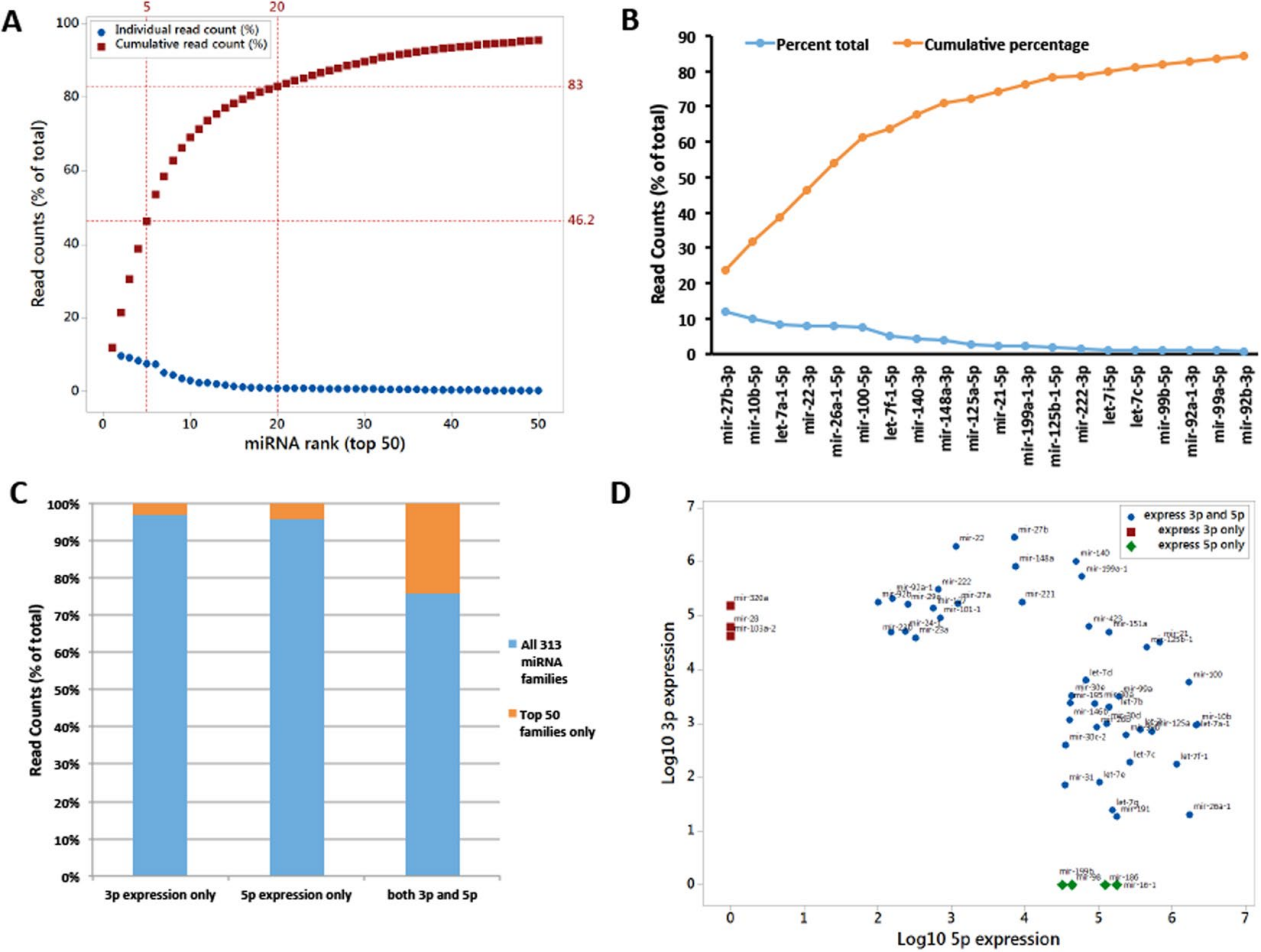
Abundance and cumulative contribution of 50 top-expressing miRNAs in primary human chondrocytes. (**A**) miRNAs were ranked according to their abundance and 50 top-ranked miRNAs were plotted against their % normalized read counts (blue dots). Cumulative contributions of the individual top 50 miRNAs are shown as brown squares. (**B**) Top 20 miRNAs that showed more than 80% cumulative expression are shown with their names. (**C**,**D**). Expression of 5p or 3p arm of individual miRNAs. (**C**). Fraction of 5p and 3p arm expression among all the 313 families that were expressed in primary human chondrocytes versus 50 top-ranked miRNAs. (**D**) Comparison of 5p and 3p expression among 50 top-ranked miRNAs expressed in primary human chondrocytes. Three miRNAs showed expression of their 3p arm only and four miRNAs expressed their 5p arm only. Rest miRNAs expressed both arms.

When miRNAs were ranked according to their abundance across all the samples, 97 top-ranked miRNAs showed 99 percent contribution (Supplementary Table 1), 50 top-ranked miRNAs showed 95.7% contribution (Fig. 1A, Supplementary Table 1) and 20 top-ranked miRNAs showed 83.6% contribution to the total pool of the chondrocyte-expressed miRNAs (Fig. 1A and B, Supplementary Table 1). miR-27b-3p was the most abundant miRNA in these samples followed by miR-10b-5p and let-7a-1-5p with a percent contribution of 11.82, 9.9 and 8.4, respectively (Fig. 1B, Supplementary Table 1). These data show that only a handful of miRNAs contribute to the total pool of miRNAs expressed in human chondrocytes. Next, we analyzed the abundance of miRNAs based on their origin in the precursor hairpin, either 5p arm or 3p arm (formerly known as main arm and the * arm). We found that among all the 313 miRNAs expressed, most of them had either 5p or 3p arms expressed, but the top 50 miRNAs had higher proportion of miRNAs expressing both the arms (Fig. 1C). Comparison of 5p and 3p expression among 50 top-ranked miRNAs found in primary human chondrocytes demonstrated that three miRNAs, miR-320a, miR-28 and miR-103a-2, showed expression of their 3p arm only and four miRNAs, miR-199b, miR-98, miR-186 and miR-16-1, expressed their 5p arm only. For rest of the miRNAs, both the arms were expressed (Fig. 1D).

### Sequence variation/isomiR profile of primary human chondrocyte miRNome

**Figure 2:**
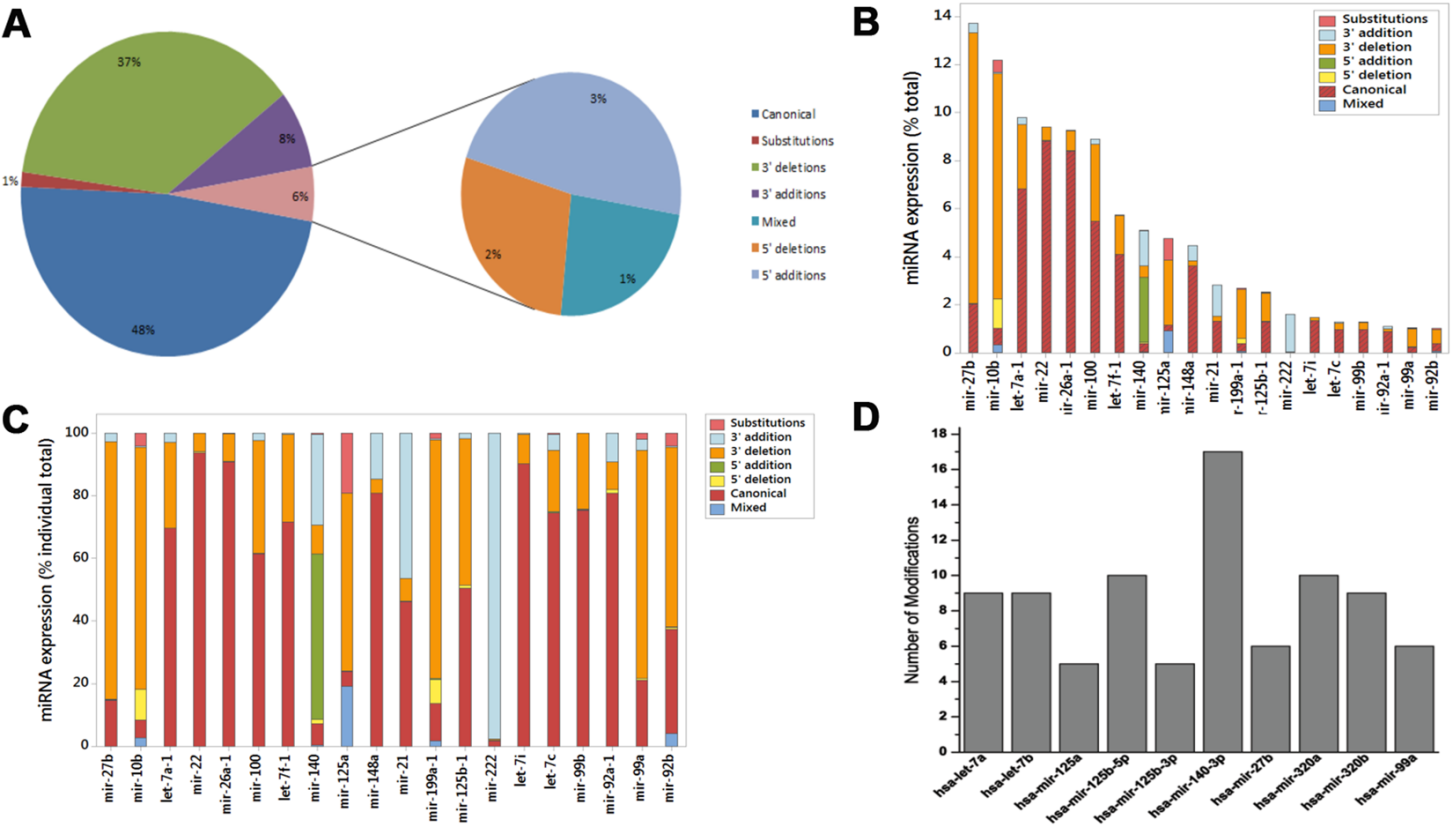
isomiR profile of miRNAs expressed in primary human chondrocytes. (**A**) Overall contribution of the canonical and seven different kinds of isomiR sequences to the total expressed repertoire of miRNAs. (**B**,**C**) isomiR expression of individual 20 top-ranked miRNAs. **B** shows the expression of isomiRs of individual miRNAs as percentage of total expression of all miRNAs. **C** shows the expression of different isomiRs of individual miRNAs as percentage of individual total. (**D**) Number of modifications in ten most modified miRNAs with ≥100 read counts.

Next, we studied the abundance of sequence isoforms (isomiRs) in the human chondrocyte miRNome. Earlier next generation sequencing based studies have reported a high degree of sequence variations in the majority of miRNAs^6,15^. Based on the types of sequence variations the isomiRs can be divided into seven categories: (i) canonical miRNAs, (ii) substitution isomiRs, (iii) 3′ deletion isomiRs, (iv) 3′ addition isomiRs, (v) 5′ deletion isomiRs, (vi) 3′ addition isomiRs and (vii) ‘mixed’ type isomiRs, with sequence changes of combinations of the prior categories. Figure 2A shows the overall contribution of the canonical miRNAs and seven different kinds of isomiR sequences to the total expressed repertoire of miRNAs. In our data set, 48% of the sequences matched with the canonical sequences reported in the miRBase, while 52% of the sequences carried some kind of variation. Among all the isomiRs, 86.5% of isomiRs had 3′ variations and a majority of them had 3′ deletions (71.1% vs 15.4 of 3′ additions). 5′ addition and 5′ deletion contributed 3% and 2%, respectively. Mixed and substitution isomiRs were only 1% each in our data set (Fig. 2A).

Next, we focused on the sequence variations among the 20 miRNAs that were top-ranked based on their overall expression in the primary human chondrocytes. Figure 2B shows the expression of isomiRs of individual miRNAs as percentage of total expression of all miRNAs. Figure 2C shows the expression of different isomiRs of individual miRNAs as percentage of individual total. For miR-27b, miR-10b, miR-199a-1 and miR-99a most of the miRNA pool was contributed by 3′ addition isomiRs. A limited number of miRNAs showed significant levels (>10%) of 3′ addition isomiRs, namely, miR-140, miR-148a, miR21, and miR-92a-1. Interestingly, miR-222 had most (97%) of the sequences in the form of 3′ addition isomiR. Only miR-125a showed >10% of both mixed type isomiRs and substitution isomiRs. Surprisingly, 5′ addition isomiR was limited to miR-140 only and accounted for 54% of the total miRNA pool of miR-140. A significant level of 5′ deletion was only specific to miR-10b and miR-119a-1. These two modification types lead to a change in seed shift resulting in change in the binding specificity of the miRNAs. Among the top 20 expressed miRNAs only 50% of the miRNAs had their canonical form as their major expressed isoform and these were let-7a-1, miR-22, miR-26a-1, miR-100, let-7f-1, miR-148a, let-7i, let-7c, miR-99b and miR-92a1. These data show the specific nature of miRNA modifications suggesting the action of different kind of sequence modifying mechanism(s) acting on different miRNAs.

Figure 2D shows the number of different isoforms present in ten most modified miRNAs with ≥100 read counts. Interestingly, miR-140-3p, that has been shown previously as the most abundant miRNA expressed in un-passaged primary human chondrocytes^16^ and miR-140 locus has been shown to have significant impact on OA development^17^, showed the maximum number of isoforms (17 in total).

### IsomiRs of miR-140-3p and effect of sequence variation on its function

**Figure 3:**
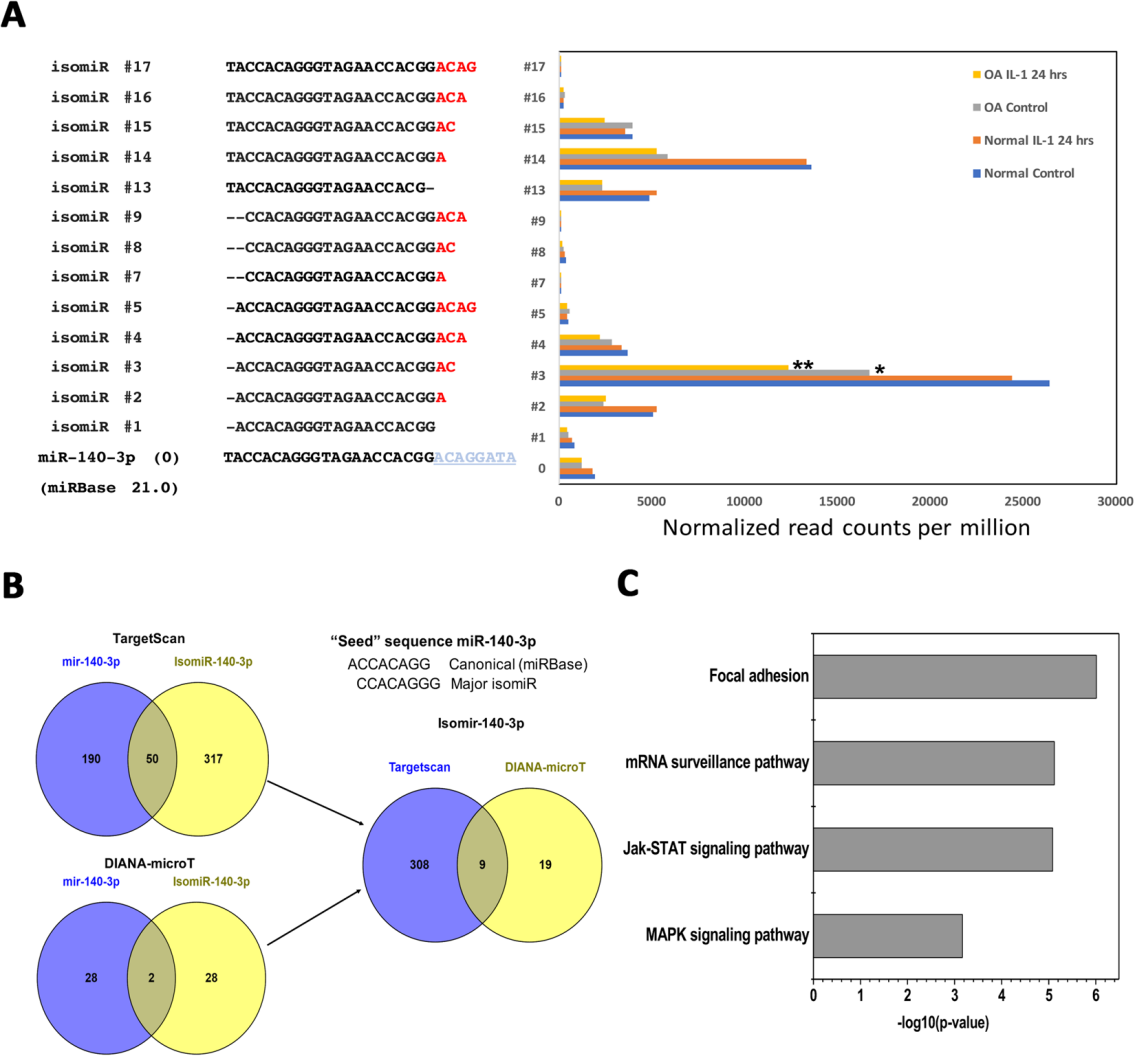
isomiRs of miR-140-3p and effect of sequence variation on its function. (**A**) Individual modifications with ≥100 read counts in miR-140-3p aligned with the archetype miRBase sequence. Reads were compared using two-tailed Student’s t-test. *p < 0.05; **p < 0.01 compared to the normal control chondrocytes. (**B**) The targeting potential of the canonical miRNA versus the most abundant isomiR (#3) of miR-140-3p was compared using TargetScan and DIANA-microT tools. The seed sequences of the two variants are shown to highlight the “seed shift”. (**C**) Pathway enrichment analysis using miRPath v.3/Diana tools shows the pathways potentially affected due to the change in targeting potential of the canonical miRNA-140-3p and isomiR#3. TarBase v7.0 and TargetScan algorithms were used to identify the targets of the miRNAs.

Since miR-140-3p showed the highest number of variants in our data set, we further focused on the analysis of the sequencing results of this miRNA. Figure 3A shows the individual modifications (isomiR #1 through #17) with ≥100 read counts in miR-140-3p aligned with the archetype miRBase sequence (#0). IsomiR #3 with a 5′ deletion and with two nucleotide additions at the 3′ end had the highest expression followed by isomiR#14, #2, #4, #13 and #14. Based on the expression level, the canonical miR-140-3p was ranked 7^th^ among all the sequence variants of this miRNA. In fact, the read count for its predominant 5′ isomiR (isomiR#3) was about 25 times higher than the canonical sequence. In addition, isomiR#3 was also found to be differentially expressed in normal samples versus samples obtained from OA patients and control cells versus IL-1β treated OA cells, while the canonical form did not show any differential expression among different conditions.

### AGO2-associated miRNAs and isomiRs in primary human chondrocytes

**Figure 4:**
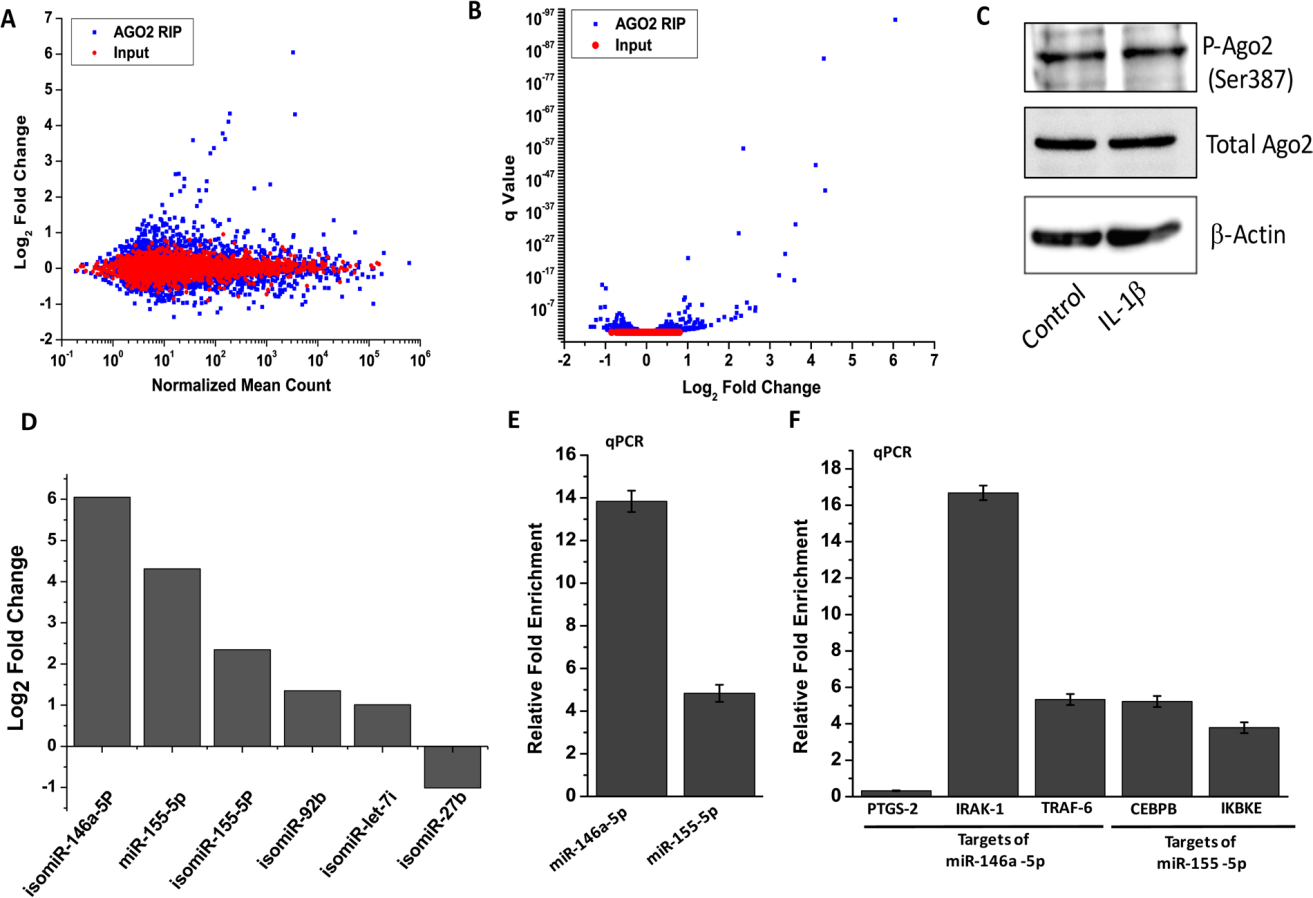
Characterization of the miRNome differentially associated with the AGO-2 complex in response to IL-1β treatment in primary human chondrocytes. Primary human chondrocytes were left untreated or treated with IL-1β (5 ng/ml) for 16 hrs followed by RNA immunoprecipitation using AGO-2 antibody (AGO2-RIP). The eluted RNA was used for library preparation using small RNA library preparation kit (Illumina) and next generation sequencing on Illumina platform. 10% input RNA was sequenced in parallel. Data were analyzed using Illumina small RNA app available on BaseSpace. (**A**) M-A plot showing the differential abundance of the miRNAs/isomiRs associated with AGO2 (blue dots) compared to input RNA (red dots). (**B**) Volcano plot showing the q Value of differentially expressed miRNAs/isomiRs vs Log2 Fold change. (**C**) Western blot analysis showing the expression and phosphorylation of the AGO2 protein in response to IL-1β. (**D**) Fold change in the expression of 6 top ranking miRNAs/isomiRs as per the data obtained by NGS. (**E**) The NGS data shown in D were validated by TaqMan real time PCR using the primers available for canonical sequences only. (**F**) Enrichment of the selected targets of miR-146a-5p and miR-155-5p in the AGO2 fractions prepared from control cells and IL-1β treated cells was quantified by real time analysis. Data are presented as relative fold enrichment of the selected mRNAs in the AGO2 fraction isolated from IL-1β-treated chondrocytes compared to untreated chondrocytes.

Next, in order to reveal which miRNAs are part of the miRNA-induced silencing complex (miRISC) and thus the truly functional miRNAs and isomiRs in primary human chondrocytes, and whether this phenomenon is affected by treatment with IL-1β, we performed RIP-Seq analysis using AGO2-specific antibody. Total RNA samples from control or IL-1β-treated primary human chondrocytes from two OA patients were used for RNA immunoprecipitation followed by next generation sequencing. The M-A plot in Fig. 4A shows the differential abundance of the miRNAs and isomiRs associated with AGO2 compared to input RNA. This analysis shows that only a limited number of miRNAs/isomiRs were differentially associated with miRISC in response to IL-1β treatment. As shown in Fig. 4B, the differential expression of those miRNAs was highly significant. Previous studies have shown that AGO2 is phosphorylated at Ser-387 by p38 MAPK (mitogen-activated protein kinase)^18^. Since p38 MAPK is activated by IL-1β in primary human chondrocytes^19^, we next checked the effect of IL-1β treatment on AGO2 expression and its phosphorylation at Ser-387. As shown in Fig. 4C, Western blot analysis revealed that the highly significant differential association of the miRNAs with AGO2 was not associated with any change in expression or phosphorylation of the AGO2 protein in response to IL-1β. Figure 4D shows the fold change in the expression of 5 top ranking miRNAs/isomiRs based on the copy number obtained by next generation sequencing (NGS). Only those miRNAs were selected that were expressed at >1000 read count and showed a log_2_ fold change of ≥+1 or ≤−1 and showed a significant differential expression. In order to validate the NGS data shown in Fig. 4D, we focused on two most well-studied miRNAs linked with the IL-1β signaling pathway, miR-146-5p and miR-155-5p, and tested their binding to AGO2 by TaqMan real time assay using the primers available for their canonical sequences (Fig. 4E). The qPCR data showed about 14-fold enrichment of miR-146a-5p and about 5-fold enrichment of miR-155-5p in the AGO2-bound fraction of IL-1β-treated chondrocytes compared to control cells. Our qPCR data matched well with our results obtained by next generation sequencing. To assess the physiological relevance of these findings we next tested whether the targets of miR-146a-5p and miR-155-5p that are also known to be involved in chondrocyte function were differentially enriched in the AGO2 complex in response to IL-1β. Data presented in Fig. 4F show that indeed all the selected targets of miR-146a-5p and miR-155-5p, except PTGS-2, were strongly enriched in the AGO2 fraction of IL-1β-treated chondrocytes compared with untreated cells, as quantified by real time analysis (Fig. 4F). Complete set of AGO2-RIP-Seq data is presented in Supplementary Table 4.

## Discussion

In this study, using next generation sequencing technology, we sought to establish a comprehensive profile of miRNAs and isomiRs expressed in primary human chondrocytes as well as those loaded on the silencing complex via binding to AGO2. Using the Illumina platform, we sequenced libraries from three OA patients and two normal subjects for general cellular expression of miRNAs and from two OA patients for AGO2-RIP-seq analysis. We prepared multiple libraries from each subject, using chondrocytes untreated or treated with IL-1β for different time points. An earlier study that reported data from small RNA sequencing analysis on human chondrocytes was performed on samples from 3 OA patients and did not include normal subjects and it mainly focused on novel miRNAs and did not include analysis of isomiRs or AGO2 binding^20^.

The data presented here showed that only a handful of miRNAs are highly expressed in chondrocytes and contribute to the majority of the miRNA pool. Out of a total of 313 miRNAs detected in primary chondrocytes, twenty miRNAs represented about 85% of the pool while only fifty miRNAs contributed to more than 95% of all the chondrocyte-expressed miRNAs. A number of the top expressing miRNAs, such as miR-27b-3p and miR-140-3p, have been extensively studied in the context of chondrocyte function and OA pathogenesis^17,21^. But the role of many other miRNAs, such as miR-100 and miR-99a, that we and Crowe *et al*. found to be highly expressed, have not been studied^20^. These studies open new avenues for research on the role of these new miRNAs found to be highly expressed in chondrocytes in cartilage homeostasis and OA pathogenesis. Even though Crowe *et al*.^20^ used a different set of adaptors, called high definition adapters, to avoid bias in sequencing reaction, there are eleven miRNAs that were common among 20 top expressing miRNAs in our samples (Table 1) and the data shown in Crowe *et al*.^20^, though there were some differences in their rankings. Some of the other miRNAs well known to play significant roles in chondrocyte physiology and disease conditions, such as miR-9, shown to regulate the expression of IL-6^22^, and miR-145, that directly inhibits the cartilage master regulator SOX9^23^ etc., were expressed at low levels, highlighting a probable bias linked with the sequencing method and due to the fact that those miRNAs despite being expressed at very low levels may have strong effects on the cellular physiology.

**Table 1:** Top 20 chondrocyte-expressed miRNAs, their relevance in chondrocyte/cartilage biology and differential AGO2 binding

Rank	miRNA	Rank in Crowe *et al*.	Role in chondrocyte/cartilage/OA known? (Ref.)	AGO2-RIP-Seq Log2Fold (Control vs IL-1β), Q-value
1	mir-27b-3p	11	Yes^21^	−1, 1.43E-08
2	mir-10b-5p	2	No	NS
3	let-7a-5p	9	No	NS
4	mir-22-3p	—	Yes^43^	NS
5	mir-26a-5p	5	Yes^48^	NS
6	mir-100-5p	14	No	NS
7	let-7f-5p	18	No	NS
8	mir-140-3p	1	Yes^20^	NS
9	mir-148a-3p	—	Yes^13^	NS
10	mir-125a-5p	—	No	NS
11	mir-21-5p	15	Yes^13^	NS
12	mir-199a-3p	—	No	NS
13	mir-125b-5p	12	Yes^13^	NS
14	mir-222-3p	—	Yes^49^	NS
15	let-7i-5p	—	No	1.01, 1.12E-23
16	let-7c-5p	17	No	NS
17	mir-99b-5p	20	No	NS
18	mir-92a-3p	—	Yes^50^	0.94, 4.87E-07
19	mir-99a-5p	—	Yes^51^	NS
20	mir-92b-3p	—	No	1.35, 3.31E-09

NS, non-significant.

Based on data from several large-scale miRNA sequencing studies, it has been proposed that the arm selection for the processing of the dominant mature miRNA can provide a mechanism to evolve the function of a particular miRNA and can be specific to tissue type and the time of developmental process or a disease condition^24–26^. We did not observe any significant change in arm selection in human chondrocytes due to the disease condition or in response to the treatment of chondrocytes with IL-1β. But, there was a significant difference in the selection of 5p arm or 3p arm between highly expressed miRNAs as compared to all the miRNAs together (Fig. 1C).

Further, for the first time, we report a detailed repertoire of isomiRs expressed in human chondrocytes isolated from both normal subjects as well as OA patients. We show the expression of a vast range of miRNA variants expressed in human chondrocytes. In fact, majority (52%) of the total miRNA pool was contributed by isomiRs (Fig. 2A). With the help of high throughput sequencing techniques, many recent studies have shown the presence of miRNA sequence variants or isomiRs in a variety of cell and tissue types^27–29^. These sequence variants are thought to be generated from a single miRNA coding locus through the imprecise editing activities of the members of the miRNA maturation and processing machinery, including Drosha and Dicer^30,31^. Apart from the ‘errors’ introduced by Drosha and Dicer, non-template nucleotide additions, especially on the 3′ end, have been reported that can also change the ends of the miRNAs, thus, affecting the stability, loading into the miRISC and targeting characteristics of the miRNAs^32,33^. Several studies have shown that these isomiRs are as active and functional as the canonical miRNAs^34,35^. Cloonan *et al*. have argued that the presence of multiple forms of a single miRNA provides it a higher on-target to off-target ratio in targeting a particular cellular pathway compared to just increasing the dosage of that particular miRNA^27^. Fine tuning of the mRNA targeting ability of a particular miRNA based on the cellular requirements may be another evolutionary benefit of the generation of isomiRs. The later point is supported by various studies in which a shift was seen in the abundance of one type of isomiR in response to a stimulus or disease condition such as bacterial infection and certain types of cancer^28,36^. In our study, we did not find any significant change in the expression of most of the isomiRs, both based on the origin of the chondrocytes, from normal subjects or OA patients, and upon treatment with IL-1β except for certain isoforms of miR-140-3p (discussed later).

Similar to other cell and tissue types^29^, human chondrocytes also showed a higher expression of 3′ addition or deletion isomiRs as compared to 5′ addition or deletion isomiRs (46% vs 6%, respectively). This may be due to the binding of AGO2 and other proteins preferentially to the 5′ end of the miRNAs protecting them from the action of nucleases and other RNA-modifying enzymes. In addition, the changes on the 5′ end will result in shift in seed sequence and will have larger impact on the targeting characteristics of the miRNA compared to changes on the 3′ end. Interestingly though, among our top 20 miRNAs, miR-10b, miR-140 and miR-199a had a high percentage of 5′ modifications compared to other miRNAs, showing the specific nature of these modifications.

miRNA-140 is the most studied microRNA to date with respect to chondrogenesis, cartilage homeostasis and the development of OA^13,37^, so much so that it is now being called as cartilage/chondrocyte-specific microRNA. Miyaki *et al*. reported that knocking out microRNA-140 in mice led to a higher predisposition to the development of age-related OA-like changes and increased cartilage destruction in surgically-induced OA^38^. Conversely, cartilage-specific overexpression of miR-140 resulted in protection from antigen-induced arthritis^38^. Our results showed several interesting aspects of the expression of miR-140 locus in primary human chondrocytes. First, miR-140-3p instead of −5p was the major expressed arm with 20-fold higher expression compared to miR-140-5p. Interestingly, Crowe *et al*. found miR-140-3p to be the most abundant microRNA among all the chondrocyte-expressed microRNAs. Second, miR-140-3p showed the highest number (n = 17) of isomiRs and many of which showed higher expression compared to the archetype sequence. A number of those isomiRs were of 5′-deletion type resulting in seed modification. In fact, seed-modifying variations were over-represented in miR-140-3p compared to the global scenario. In silico analysis to check the effect of the seed-modifying 5′-deletion in isomiR#3 predicted a significant change in the putative mRNA targets of miR-140-3p. Third, some of the isomiRs of miR-140-3p (e.g. isomiR#3 in Fig. 3) showed differential expression in disease condition as well as upon treatment with IL-1β. Fourth, along with being expressed at high levels in primary chondrocytes, miR-140-3p and its isomiRs were among the highly enriched miRNAs in these cells but did not show a differential enrichment in cells treated with IL-1β compared to untreated controls. This highlights a constitutive role of miR-140-3p and its isomiRs in the maintenance and homeostasis of human chondrocytes.

We also tested whether 5′ deletion in isomiR#3 resulting in the seed shift of the canonical miR-140-3p will impact its binding ability to its target mRNA sequences. Figure 3B shows the comparison of the targeting potential of the canonical miRNA versus the most abundant isomiR (#3) of miR-140-3p using TargetScan and DIANA-microT tools. Both TargetScan and DIANA-microT analyses show that the putative targets of the two variants are very different. TargetScan predicted a total of 190 unique targets of canonical miR-140-3p and 317 unique putative targets of isomiR#3 (Fig. 3B). Only 50 targets were shared between the two isoforms. DIANA-microT predicted a total of 28 unique putative targets for both the isoforms and two targets that were common between the two isoforms (Fig. 3B). Figure 3C shows the pathway enrichment analysis using miRPath v.3/Diana tools highlighting the pathways potentially affected due to the change in targeting potential of the canonical miRNA-140-3p and isomiR#3.

Similar to our results, Krawczynski *et al*. reported miR-140-3p as the miRNA showing the highest number (n = 14) of isoforms in the pig endometrium^39^. The bio-informatic analysis in their study revealed significant differences in the targeting characteristics of their dominant miR-140-3p 5′ DEL U isomiR, that had the same nucleotide sequence as our dominant isomiR#3. Their validation study by forced expression of this isoform in primary porcine stromal cells showed a significant difference in the silencing of the genes predicted to be the target of this isoform compared to the canonical sequence^39^.

miRISC loading via binding to one of its components, AGO2, is considered an indicator of a miRNA’s functional nature. To test this in our chondrocyte samples isolated from two OA patients, we explored the loading of miRNAs and isomiRs on AGO2 protein and checked whether it changes in response to IL-1β treatment. AGO2 RIP-Seq is an established technique used to comprehensively identify the miRNAs and mRNAs loaded to the miRNA silencing complex at high resolution. This technique has been recently applied to analyze the functional miRnome and miRNA targetome in other cell/tissue types^40–42^. We utilized a monoclonal antibody highly specific to human/mouse AGO2 to purify the miRISC along with all the bound miRNAs, isomiRs and mRNAs from untreated and IL-1β-treated primary human chondrocytes. We then eluted the miRNAs from the complex and purified using the column binding technology. The purified small RNA fraction was then used to generate libraries. Subsequently, we size-selected the libraries to enrich for miRNA-specific libraries. This experiment helped us generate, for the first time, a comprehensive profile of miRISC-bound miRNAs and isomiRs in primary human chondrocytes. In addition, we found that the treatment of human chondrocytes with IL-1β dramatically changed the global binding profile of miRNAs and isomiRs to the miRNA silencing complex via AGO2 without significantly affecting their total cellular expression (Fig. 4A and B). Amongst the miRNAs that showed high differential AGO2 binding, a 3′ del T isomiR of miR-146a-5p showed the highest enrichment in IL-1β-treated chondrocytes compared to untreated controls. These data are in line with the known role of miR-146 in inflammation, IL-1β signaling, OA pathogenesis and OA-associated pain^43,44^. The second and third top enriched miRNAs were canonical miR-155-5p and its 3′ del T isomiR. Together with miR-146a, miR-155 too has been implicated in the cellular response to IL-1β and in inflammatory pathways^37,45^. A 3′ diadenylated isoform of miR-27b-3p was the only high expressing miRNA (>1000 mean read count) that showed a significantly lower enrichment in IL-1β-treated chondrocytes compared to untreated control cells (log_2_ fold change, −1; p-value, 1.43E-08). The role of miR-27b in chondrocyte function and OA pathogenesis has been previously studied^21,46^. Our present study further corroborates those observations and highlights the significant physiological roles of miR-27b-3p in chondrocytes. To the best of our knowledge this is the first study to date in any cell type whereby a microRNA and isomiR profile was revealed showing the differential enrichment of certain miRNAs in response to IL-1β.

In conclusion, here we have presented the data from deep sequencing and AGO2-RIP-seq studies that reveal the complex nature of miRNA function in human chondrocytes. We show that miRNAs expressed in chondrocytes are extensively modified, in many cases the modified sequences show higher expression than the canonical sequences and these modified isoforms are also functional as evident from their binding to the miRISC. These data also revealed the profile of those miRNAs differentially enriched in the silencing complex in response to IL-1β. Additionally, this study opens new avenues for future detailed investigations of the role of miRNA function in the pathogenesis of diseases such as OA where chondrocytes and cartilage are affected.

## Methods

### Human Cartilage samples and chondrocytes preparation

The study protocol was reviewed and approved by the Institutional Review Board (IRB) of Northeast Ohio Medical University, Rootstown, OH, as a “non-human subject study under 45 CFR” and that no informed consent was needed. All the experiments were performed in accordance with relevant guidelines and regulations and as approved by the IRB. Normal articular cartilage samples collected from post-mortem donors were procured from the National Disease Research Interchange (NDRI, Philadelphia, PA). OA cartilage samples were collected from patients who underwent total joint replacement surgery at Summa St. Thomas Hospital (Akron, OH). Cartilage was resected from macroscopically unaffected areas and chondrocytes were prepared as previously described^22^. Chondrocytes were grown in Dulbecco’s modified Eagle’s medium (DMEM) supplemented with 10% Fetal Calf Serum (FCS), 100 U/ml Pennicillin and 100 mg/ml Streptomycin for 2–3 days after plating and only primary (unpassaged) cells were used in the experiments. At about 80% confluence, chondrocytes were serum starved overnight and were then treated with IL-1β (2 ng/ml).

### Argonaute-2 RNA-immunoprecipitation (AGO2-RIP)

miRISC-associated miRNome was purified by RNA immunoprecipitation using microRNA isolation kit, following the manufacturer’s protocol (Wako Pure Chemical Industries, Osaka, Japan, Cat. No. 292-66701). Briefly, following the treatment 10 million primary human chondrocytes were lysed in the lysis buffer provided with the kit. The cleared lysate was mixed with the AGO-2 beads for 2 hrs at 4 °C with rotation. The beads were washed with the wash buffer for 3 times and then eluted. The immunoprecipitated RNA was then purified using the columns provided with the kit that was then used to prepare the libraries for the next generation sequencing.

The global sequencing data were plotted as M-A plot and volcano plot. The M-A plot shows the log ratios of the read counts of IL-1β-treated samples versus the control samples on the y-axis and the average of the read counts on a log scale on the x-axis. Each dot represents an individual miRNA. The volcano plot shows the q-values (p-values adjusted for multiple parameters) of differential expression for individual miRNAs on the y-axis versus the fold change on x-axis.

### RNA extraction

Total RNA was extracted from the chondrocytes using the miRNeasy Kit (Qiagen). RNA was quantified using Qubit 2.0 Fluorometer (Thermo Fisher Scientific) and the integrity was evaluated using an RNA 6000 Nano chip on Agilent Bioanalyzer 2100 (Agilent Technologies). RNA samples with an RNA Integrity Number (RIN) of > 8 were selected for library preparation.

### Library preparation and next generation sequencing

Small RNA libraries were constructed using the TruSeq small RNA library prep kit (Illumina), following the manufacturer’s protocol. Briefly, 1 μg of total RNA from each sample was ligated to 3′ adaptor using T4 RNA Ligase 2 deletion mutant (Epicentre) followed by ligation to 5′ adaptor using T4 RNA ligase (Illumina). The ligated fragment was reverse transcribed followed by PCR amplification (11 cycles). The amplified products were size fractionated on 6% Novex TBE PAGE gels (Life Technologies). A band corresponding to 145–150 bp was purified and denatured. Sequencing was performed on Illumina MiSeq platform using MiSeq reagent kit v2.

### Computational analysis of sequencing data and isomiR identification

Sequencing reads were analyzed using the CLC Genomics Workbench ver. 8.5.1 (http://www.clcbio.com/products/clc-genomics-workbench/). Briefly, the reads were trimmed to remove adapter sequences. Low quality reads and reads smaller than 16 nucleotides were discarded. Trimmed reads were mapped to human mature and precursor miRNAs using the miRBase version 21^47^ (http://www.mirbase.org/). The number of reads mapped to each mature miRNA was counted, and then normalized to account for differences in sequencing depth by using counts-per-million (CPM). To evaluate miRNA sequence variability, trimmed and mapped reads were analyzed for sequence changes, including 5′ and 3′-end modifications. Specifically, we focused on 3′ addition variants derived through adenylation or uridylation mechanisms (i.e., As or Us additions).

### Data Availability

All data generated or analysed during this study are included in this published article (and its Supplementary Information files).

## Electronic supplementary material

Dataset 1

Dataset 2

Dataset 3

Dataset 4
